# Innovations in Gerontology Education

**DOI:** 10.1093/geroni/igab046.559

**Published:** 2021-12-17

**Authors:** Kimberly McDonald, Jennifer Ellis

**Affiliations:** 1 Northwood Technical College, New Richmond, Wisconsin, United States; 2 Northwood Technical College, Superior, Wisconsin, United States

## Abstract

This session examines innovative approaches to effectively address the aging services workforce needs in rural, Midwestern settings. Presenters will explore career pathways and transfer opportunities in gerontology education, as well as best practices for addressing the educational, professional and personal needs of diverse student populations.

